# Multiple mini interview (MMI) for general practice training selection in Australia: interviewers’ motivation

**DOI:** 10.1186/s12909-018-1128-z

**Published:** 2018-01-25

**Authors:** Annette Burgess, Chris Roberts, Premala Sureshkumar, Karyn Mossman

**Affiliations:** 1 Sydney Medical School – Education Office, The University of Sydney, Edward Ford Building A27, Sydney, NSW Australia; 2 Sydney Medical School – Concord, The University of Sydney, Sydney, NSW Australia; 3 Sydney Medical School – Northern, The University of Sydney, Sydney, NSW Australia

**Keywords:** Multiple mini interview, Admission, Selection, Specialty training, General practice, Assessment

## Abstract

**Background:**

Multiple Mini Interviews (MMIs) are being used by a growing number of postgraduate training programs and medical schools as their interview process for selection entry. The Australian General Practice and Training (AGPT) used a National Assessment Centre (NAC) approach to selection into General Practice (GP) Training, which include MMIs. Interviewing is a resource intensive process, and implementation of the MMI requires a large number of interviewers, with a number of candidates being interviewed simultaneously. In 2015, 308 interviewers participated in the MMI process – a decrease from 340 interviewers in 2014, and 310 in 2013. At the same time, the number of applicants has steadily increased, with 1930 applications received in 2013; 2254 in 2014; and 2360 in 2015. This has raised concerns regarding the increasing recruitment needs, and the need to retain interviewers for subsequent years of MMIs. In order to investigate interviewers’ reasons for participating in MMIs, we utilised self-determination theory (SDT) to consider interviewers’ motivation to take part in MMIs at national selection centres.

**Methods:**

In 2015, 308 interviewers were recruited from 17 Regional Training Providers (RTPs) to participate in the MMI process at one of 15 NACs. For this study, a convenience sample of NAC sites was used. Forty interviewers were interviewed (*n* = 40; 40/308 = 13%) from five NACs. Framework analysis was used to code and categorise data into themes.

**Results:**

Interviewers’ motivation to take part as interviewers were largely related to their sense of duty, their desire to contribute their expertise to the process, and their desire to have input into selection of GP Registrars; a sense of duty to their profession; and an opportunity to meet with colleagues and future trainees. Interviewers also highlighted factors hindering motivation, which sometimes included the large number of candidates seen in one day.

**Conclusion:**

Interviewers’ motivation for contributing to the MMIs was largely related to their desire to contribute to their profession, and ultimately improve future patient care. Interviewers recognised the importance of interviewing, and felt their individual roles made a crucial contribution to the profession of general practice. Good administration and leadership at each NAC is needed. By gaining an understanding of interviewers’ motivation, and enhancing this, engagement and retention of interviewers may be increased.

**Electronic supplementary material:**

The online version of this article (10.1186/s12909-018-1128-z) contains supplementary material, which is available to authorized users.

## Background

Traditionally, systems of ranking candidates for selection into specialty training programs have consisted of a mixture of application letter, panel interviews, and references [1]. However, there has been global recognition of the need for more robust systems in regard to underlying assessable constructs, psychometrics, fairness and defensibility. Assessment centres are now used as a model where candidates attend a venue to undertake one assessment or more for the purpose of selection into postgraduate training [2]. Assessments commonly include situational judgement tests (SJT) [3] and the multiple-mini-interview (MMI) [4].

Multiple Mini Interviews (MMIs) are being used by a growing number of postgraduate training programs and medical schools as their interview process to select entry into their training programs. Canada, Australia and the United Kingdom have used MMI to assess non-cognitive characteristics of postgraduate medical trainees, with early findings suggesting that the MMI offers a useful format to select junior doctors for specialty training [5–9]. The expectation is that this method will assist in the selection of candidates who are team players, with communication skills that allow them to connect with both patients and other healthcare professionals. The MMI is different from the typical interview process in a number of ways. Applicants spend a brief period of time with one interviewer (on a focused question) and then move to the next interview room (for another distinct interaction). The MMI format is based on the Objective Structured Clinical Examination (OSCE) format [5, 8]. The MMI format shows greater reliability and content validity than the traditional interviews ([5] and is more cost-effective [10].

Setting up the MMI is a resource intensive process, and implementation requires a large number of interviewers, with a number of candidates being interviewed simultaneously. Interviewers are typically medical practitioners; this requires them to be taken away from patient care in order to participate in the MMI. Given the extensive resource requirements and recruitment requirements of MMIs, the question arises as to what motivates practitioners to take part in them. Although motivation is a complex issue that can be theorised in a number of ways, developing an understanding of the interviewers’ motivation to take part in the MMI selection process is important to future recruitment and retention of interviewers. Although several studies have considered candidates’ and interviewers’ perceptions of fairness [9, 11], little has been published regarding interviewers’ motivation to participate in the process. The context of our study was the MMI process used in the National Assessment Centre (NAC) for entry into Australian General Practice training. Our research question was “What motivates individuals associated with Regional Training Providers (RTPs) to participate as interviewers in a national assessment selection process?”

### Theoretical framework

Interviewers’ motivation can be viewed through the lens of self-determination theory (SDT) [11]. Although motivation can be considered in a number of ways, SDT, developed by Deci & Ryan (2000) was applied [12]. SDT proposes that for individuals to be intrinsically motivated, three key elements are needed: 1) autonomy; 2) competence and 3) relatedness. Autonomy relates to an individual’s sense of choice in what they are doing, and their own aspirations [12]. Competence relates to an individual’s desire to attain proficiency in an area [12]. Relatedness refers to a sense of connectedness with others with similar goals and purpose [12]. We utilised SDT as a theoretical framework to explore interviewers’ motivation to take part in the MMI selection process.

## Methods

### Research context

The context for our research was the 2015 NAC MMI process. A total of 2197 eligible applicants were assessed in the NAC process and 2154 applicants completed the MMI. Of the 2197 applicants assessed that year, there were a greater proportion of females (*N* = 1301; 59%) than males (*N* = 896; 41%).

#### Interviewers

In 2015, 308 interviewers were recruited by invitation from 17 RTPs to participate in the MMI process at one of 15 NACs. The interviewers were paid a standard rate of pay for their time, as well as travel and accommodation costs. Those invited included GPs with formal links to the RTPs, for example, those with responsibilities for training of GP registrars, and staff of the RTPs. There was a professional expectation that those with formal, senior salaried roles within the RTPs, such as the Chief Executive Officer of the RTPs, and the Directors of Training, should contribute as interviewers. Interviewers either attended a two-hour interview face-to-face training session, or completed an on-line training course. They were familiarised with the interviewing technique, the marking scheme and advice on avoiding common causes of interviewer bias. They were encouraged to make notes to justify their marking decisions.

#### MMI stations

Each circuit of the MMI consisted of six stations, with a single interviewer at each. The questions for each station were blueprinted against the six expected competency domains of entry-level registrars provided by The Royal Australian College of General Practice and the Australian College of Rural and Remote Medicine. These include communication and interpersonal skills; clinical reasoning and analytical/problem solving skills; organisational management skills; sense of vocation; personal attributes (such as the capacity for self reflection and awareness of the impact of cultural issues on delivery of primary healthcare); and ethical attributes. The MMI took place in interview format. It utilised many short independent assessments, each assessed by one trained interviewer, and each taking six minutes to complete. After each circuit or two, interviewers were typically debriefed in a short session facilitated by a senior medical educator.

### Data collection

#### Survey

As part of a systematic evaluation of the process, an anonymous interviewer questionnaire was distributed to each interviewer immediately following completion of the MMI at the NAC. The questionnaire included questions relating to interviewer demographics (age, prior training, current role), which is reported in the results.

#### Interviews

A sample of NAC sites was used to recruit interviewers from both major and smaller urban centres as well as a rural centre for interviews. In total, 40 MMI interviewers were interviewed (*n* = 40; 40/308 = 13%) from five NACs, including Melbourne, Sydney (Central), Newcastle, Adelaide and Darwin. All MMI interviewers who were interviewed were general practitioners, and were interviewed on a day that they participated as interviewers. They were invited to voluntarily take part in the study by external researchers who were not employed by the RTPs, and were interviewed by these researchers (authors AB, CR, PS or KM).

The interview questions were structured and designed to gain a deep understanding of the interviewers’ motivation to participate in the MMI interviews in addition to their usual work (Additional file 1). For example “*What factors motivated you to take part as an interviewer today?”, “Are there any elements need improving to make it more likely you would return to interview next year?”.*

### Data analysis

Interview data were transcribed verbatim. Framework analysis was used to code and categorise data into themes [13, 14]. Framework analysis provides a method to structure data to assist in answering research questions. The initial analysis was conducted by the first author on a sample of data, with the aim to identify recurrent themes in the. Emergent themes in the dataset appeared to resonate closely with key constructs of SDT [12]. Following a face-to-face meeting and discussion with all authors, it was decided that SDT would be used as a conceptual framework to identify and code recurrent themes. A coding framework was developed to code the entire dataset through the theoretical lens of SDT.

### Ethical considerations

The University of Sydney Human Research Ethics Committee approved the research. All interviewers gave consent to participate in the study, and were reassured the data was strictly de-identified to protect participant privacy.

## Results

### Interviewer demographics

Demographic information on participating MMI interviewers were collected by survey, with 226/308 (73%) responding. A third of these interviewers were aged 50–59 years and only 1% was below the age of 29 years. Fifty six percent of respondents were female, and 44% male. The majority (86%) were from an English speaking background. A third of the respondents had been in Australia for more than 10 years and 65% for their whole life. Their roles within RTPs were senior administration (*n* = 15, 7%), medical educator (*n* = 115, 51%) and supervisor (*n* = 96, 42%). The medical educators and supervisors were also qualified general practitioners. Sixty five percent (*n* = 147) of interviewers have had their primary medical qualification from Australia or New Zealand. Seven percent (*n* = 15) have had their PMQ in the UK and 7 (3%) in India. From the assessment data, interviewers conducted a total of 12,924 single MMI interviews, with a single interviewer averaging 42 interviews. Most (27.3%) interviewers sat two stations, with 13.3% of interviewers sitting all six MMI stations.

### Interview results

Forty MMI interviewers were interviewed (*n* = 40; 40/308 = 13%) from five NACs, which included urban and rural centres; Melbourne, Sydney (Central), Newcastle, Adelaide and Darwin.

Interviewer responses to questions relating to their motivation to participate in the MMIs were mapped to the conceptual framework of Self Determination theory (SDT). Using this framework, we illustrate how clinicians’ motivation to take part as interviewers were largely related to their sense of duty, their desire to contribute their expertise to the process, and their desire to have input into selection of GP Registrars; a sense of duty to their profession; and an opportunity to meet with colleagues and future registrars. Interviewers also highlighted factors hindering motivation, which included the long daily hours and the large number of candidates seen in one day.

#### Factors relating to a sense of autonomy

This theme refers to interviewers’ sense of choice and volition in participating in the MMIs [15, 16]. Many interviewers emphasised their interest in education, and their desire to be part of the complete process from selection through to training:

*“I have an interest in GP education. I’m a general practitioner for four days out of the week and I’m a supervisor of GP registrars, and also with…. international medical graduates doing community teaching visits. So this sort of completes the process of having some input at the very beginning of the process”.*

Interviewers wanted to have input into the selection process, particularly when they, themselves were involved in training:

*“My job as a medical educator involves training registrars. So very motivated to see what comes – what’s coming to us and I guess have some, influence in that – that the selection process is done properly, and that they’re being judged fairly and appropriately. So I guess given that my job involves dealing with the end result of the selection process, I’m interested that the selection process is done properly”.*

Interviewers felt they carried a responsibility to help ensure the right candidates were selected for training:

*“I think it’s really important that we select the right candidates for GP training and – because otherwise, I’m a medical educator and I’m a supervisor and otherwise we are just in all sorts of bother if we’re starting off with people who are inappropriately chosen for this speciality. So all the criteria that each of the interview questions use are important criteria for selection and I’d like to be a part of the team that selects the most appropriate people for this type of speciality”.*

Interviewers felt that by being part of the interview process, they could reduce the amount of ‘difficult’ GP registrars who required excessive remediation and improve the ‘quality’ of successful candidates:

*“A part of it, I’m doing a fair bit of work with registrar, on learning interventions and remediation. And, working with those registrars you, sort of wonder how they managed to get onto the programme in the first place because the nature of their deficits just makes it quite clear that, at least at this stage in their careers and in their life, general practice isn’t quite the right fit for them. So, I think… getting a selection process that works in the best way possible is actually vital to training. And also how resources are allocated. Those registrars, they actually… take up quite a lot of money and time and effort, which means that, for everyone else… there’s not quite so much to go around. So I know that I only get to see a small sample of registrars, but, I guess, that’s why I’ve become involved”.*

#### Factors relating to a sense of competence

This theme refers to supervisors being motivated by a sense of mastery and competence in their field of expertise.^5^ Some interviewers thought it was important that interviewers were experienced general practitioners, and felt their own expertise was beneficial to the selection process:

*“I think having someone who is an experienced GP being part of the interviewing is a good idea”.*

Supervisors reported they enjoyed learning while interviewing. Interviewers felt they learnt by participating in the debriefing meeting at the end of the MMI circuit where they reflected on their scoring of candidates and the score of their colleagues. These discussion sessions became a useful method of work-based training:

*“The dialogue with the other interviewers about the candidates, and how other colleagues think and reflected is an education for all of us”.*

Additionally, they wanted to help ensure that a fair process was followed:

**“** *I had been involved in interviews, which I’m not going to mention where or when, that I think the process has been a bit ordinary as opposed to - I’m not sure that all the interviewers really were doing it exactly the way they were supposed to be doing it. So I guess I’m really keen to make sure that it’s done, like, fairly for them and consistently and all that sort of stuff”.*

Part of the motivation of some interviewers to travel to various centres within the state to interview was to in order to gain a wider view of the standards that determine a candidates’ suitability for training. Additionally, medical educators were interested in how the logistical process was carried out by other RTPs at other NACs:

*“It is worthwhile seeing another centre. I think it’s worthwhile seeing candidates that you’re probably not going to see in your area. It’s good to see how the people run this process, ‘cause, you know, ours isn’t the only way”.*

They felt providing succinct written feedback was an obligation, and this was an important and learnt skill that required development:

“*I think the interviewers feel a very strong sense of professionalism, and then the point about that is that trying to provide in that very tight timeframe, for those more borderline candidates a succinct summary of some of the key points that are based on what was seen and heard, not necessarily making some general value judgements. It’s quite difficult. It’s quite a hard skill”.*

#### Factors relating to a sense of relatedness

This theme refers to interviewers’ being motived by the feeling of connectedness to others with similar goals and purpose.^10^ Interviewers expressed a sense of relatedness to other GP interviewers, and a common sense of duty in relation to their profession. Interviewers felt they had a professional obligation to interview.

*“Well, I think it's a professional obligation... and it's part of sharing responsibility as part of the team”.*

Particularly those who held positions as trainers and were directly employed by the RTPs felt an obligation to participate:

*“Because I work for the organisation I'm in a different position from a, GP supervisor for example who comes in who's not actually employed by the organisation. So I am obliged to take part. I don't regard that as a burden. It's part of my role really. I’m a teacher as well, so I’m interested in the progress of students and how the selection processing, so all the education process”.*

Interviewers were motivated to participate out of a sense of duty not only to their profession, but also to the candidates:

*“The selection process is important to try to get the right candidates. … and I know they have difficulty, obviously, sometimes getting enough interviewers”.*

From their own experience, interviewers were able to relate to where candidates were at in their careers, and were interested to hear about current experiences of the junior doctors:

*“I find it really interesting to interview young doctors and I find it particularly interesting to get an idea of what the – it’s almost an anthropological approach, what is life like for these doctors? You know, what are the hospitals like? What are the pressures that they’re under? How do they make these decisions?”*

Interviewers enjoyed the opportunity to meet with their colleagues at the NACs:

*“Over the years you build up a lot of… networks with other medical educators, both from the RTP and other RTP’s, and even in good old Melbourne. So, it’s a good way of doing it. We tend to get together again at exam time, and then medical educator days. So there’s a bit of a… collegiate brethren if you like”.*

Learning through practice was considered by interviewers to be an integral part of their learning experience:

*“actually doing it in practice, I think you can only really understand that by actually doing it on the day, because, you know, even, sort of – I think we went through, maybe, three or four, sort of, hypotheticals, um, but again, you don’t, sort of, really appreciate the diversity and how it all unfolds until the actual day…because, there’s so much variability in, the responses from the registrars, I – I don’t think any training will actually fully equip you for that”.*

They found their discussions with experienced interviewers, and reflection on their own reasoning provided a form of training:

*“….after a while you - you get - the language, the idea; you’re learning and reflecting on what you’re doing. I think that’s our best training”.*

#### Factors hindering motivation

Good administration and leadership at each NAC was needed. Factors hindering motivation were largely related to administrative planning and procedures on the day of the NACs. At some NAC sites, interviewers would see as many as 40 candidates per day, which most found arduous.

*“Thirty would be better; it would be easier… by the time you get to the last one you're getting pretty tired… candidates are disadvantaged if they come in the last session of the day”.*

There were differences noted between NAC sites regarding how the questions were being rotated between interviewers. In general, interviewers preferred to be allocated two different stations per day, staying in one station for half of the day, and then the other:

*“I think I would be more proficient if I stay in one station. Maybe at least for one session in the morning, and a session in the afternoon, rather than six questions during the morning and six in the afternoon, all mixed up. You’ve got to learn each station as you go on”.*

Interviewers reported that they were not always provided with a timetable for the day, which would be useful for them:

*“So I, sort of, know that morning tea - I think yesterday we were all a bit surprised, and then we thought, right. Half an hour for lunch, and then we’ve lost 15 minutes. So just having a timetable on our desks, I think, would be really useful”.*

Briefing procedures prior to the start of the MMIs were important to interviewers and were not always adhered to:

*“…we’d normally just have a quick chat beforehand with all of the interviewers with someone who’s in charge of us all, and – just talking about how the procedure will run and, um, you know, just reminding us to stick to the criteria in the – the questions that we’ve been allocated and… just a few, sort of, basic things. But it didn’t happen this time. There was no briefing for me….* just a quick briefing would be helpful*”.*

## Discussion

Our study utilised the conceptual framework of SDT to explore interviewers’ motivation to participate in the MMIs. Interviewers’ motivation to take part as interviewers was largely related to their interest in education, their sense of duty to their profession, their desire to contribute their expertise to the selection process, and their desire to have input into selection of GP Registrars. Interviewers also valued the opportunity to meet and learn from colleagues, as well as the opportunity to develop an understanding of the needs of future trainees. Lack of attention to detail in administrative preparation and implementation of the MMIs were identified as factors that may negatively impact on motivation. Although there is overlap between the three key elements of SDT theory (autonomy, competence and relatedness) [12], each is considered below in the context of interviewers feedback regarding their motivation to participate in the MMIs.

### Autonomy

Whether working alone or with others, individuals prefer to have a sense of choice [15.16]. A common theme was that the doctors liked having input into the selection of registrars. They saw their individual participation from RTPs into a national selection process to be important, particularly when they, themselves, would be involving in educating and training the successful applicants at their local RTPs. Interviewers expressed an understanding of how their contributions as interviewers might impact on general practice training, by helping to ensure that unsuitable and potentially difficult doctors are kept away from practice. Certainly, within Australia, there has been a recent increase in reported cases of unprofessional behaviour by doctors [17]. A crucial aim is to predict which candidates will continue on to become capable medical practitioners, and to dismiss those who are likely to display poor performance in future practice due to lack of clinical knowledge, and poor professional behaviour [18].

### Competence

Individuals like to feel they have a “mastery” of their profession [19]. Additionally, individuals enjoy mastering tasks in which they are engaged [12, 19]. Reflective practice offers the potential to engage and motivate individuals to even higher levels of expertise [20]. Interviewers felt interviewing and attending briefing meetings provided opportunities to practice and reinforce their interview skills. Interviewers expressed a sense of achievement in interviewing, and new challenges were presented to them. For example, providing succinct written comments when marking candidates on their performance in the MMI interviews was recognised as a learnt skill. An optimal level of challenge is important in working towards the mastery of a subject [15], and the interviewers improved their professionalism skills in interviewing.

### Relatedness

When individuals have similar goals and a similar sense of purpose, a sense of connectedness may be fostered [12, 15, 21]. Although interviewers felt a sense of duty to their profession to participate, they also valued the opportunity to network with their peers. Connectedness is fostered within groups with the same ideals and goals [12, 15, 21]. The interviewers appreciated and enjoyed being part of a like-minded professional community, interested in improving the selection process through their expertise. The learning context of the MMIs, with breaks to meet and discuss applicants’ performance involves a process of socialisation, and the social context of these meetings promoted communal engagement from experienced and less experienced interviewers. Interviewers also found the MMIs to be an opportunity to connect with the junior doctors, many of whom will be part of the next generation of general practitioners, and learn about their current challenges.

### Factors hindering motivation

Our results indicated signs of organisational overload in delivering the complex logistics within available resources. Factors hindering interviewer motivation were largely related to the need for careful attention to administrative details at some sites. Some interviewers had particular expectations regarding the organisation and implementation of the MMIs, and these expectations were not always met. It was considered that improvements were needed in terms of the number of candidates interviewed each day; the provision of timetables; and clear briefing procedures. Previous research has demonstrated that support for the needs of staff increase work satisfaction [22]. Lack of support, and lack of consideration for the needs of staff hinder motivation.

### Strengths and limitations of the study

This is the first study to look at the motivation of MMI interviewers to take part in a large scale selection process into specialty training. We acknowledge that interviewers who participated in this study had voluntarily chosen to do so, which may have biased our results. We also acknowledge that the opinions of the interviewers who participated in the study may not be representative of all interviewers. Although there was a balanced representation of quotes from individuals who were interviewed, we were unable to collect individual participant demographics because of the need to have minimal impact on timing of a high stakes assessment.

Although the use of the National Assessment Centre MMI is an annual event, we consider it important to explore interviewers’ motivation to take part, as there are multiple sites that each interviewer may attend, and retention of interviewers is an important factor. Future research might include research on training methods for interviewers and longitudinal studies that consider factors contributing to interviewer retention.

## Conclusion

Recruiting the best candidates to postgraduate medical training programs is central to the success and quality of the medical workforce. The interviewers’ motivation for contributing to the MMIs was largely related to their desire to contribute to their profession, and ultimately improve future patient care. Interviewers recognised the importance of interviewing, and felt their individual roles made a crucial contribution to the profession of general practice. Interviewers conveyed a sense of duty to their profession; a desire to contribute their own expertise, and improve their own interview skills. This study also identified key elements to maintaining the interest of interviewers and recruiting further interviewers: attention to detail in administration, with shorter days, longer breaks, and adherence to briefing procedures. By gaining an understanding of interviewers’ motivation, and implementing methods to enhance this, engagement and retention of interviewers may be increased.

## Additional file

Additional file 1: Interviewer Guide. (DOCX 14 kb)

## Abbreviations

AGPT: The Australian General Practice and Training (AGPT)
GP: General Practice
MMI: Multiple Mini Interview
NAC: National Assessment Centre
RTP: Regional Training Provider
SDT: self-determination theory
SJT: situational judgement tests
