# Supplementary material for: Multiple mini interview (MMI) for general practice training selection in Australia: interviewers’ motivation
Source: BMC Med Educ. 2018 Jan 25;18:21. doi: 10.1186/s12909-018-1128-z (PMC5785877; doi:10.1186/s12909-018-1128-z)
Supplement: Additional file 1: — Interviewer Guide. (DOCX 14 kb) [file 12909_2018_1128_MOESM1_ESM.docx]

INTERVIEWER GUIDE

- What kind of training did you receive for being an interviewer prior to the NAC? What kind of training would you have liked to receive?
- To what extent do you feel the interview training prepared you for the interviewing task? If unprepared, how could the training be improved?
- What kind of briefing did you receive on the day of the NAC? What kind of briefing would you have liked to receive?
- What motivated you to take part as an interviewer today?
- Are there any elements need improving to make it more likely you would return to interview next year?
- How were you recruited?
- Do you think the MMIs give applicants enough opportunity to display their relevant knowledge and skills?
- How do you find allocating scores to candidates?
- Did you feel as though you were restricted by the ‘boundaries’ of your question? That you found yourself wanting to ask or seek additional information but were not allowed to.
- As you were interviewing did you feel that the applicants engaged with the questions?
- What strategies did you use to elicit more information from applicants, or get them to focus in on particular aspects of the question?
- How did you feel about the pace of the day? Too slow/intense? What aspects would you change to make your day go by more smoothly?

Probes: Well organised day with break times/too much work load/was able to concentrate throughout the day/friendly and helpful NAC staff etc

- Would you like to receive feedback from the interviewers’ perspective?
- What suggestions do you have for improving the MMI process?
- Would you have liked more question variation?
- Is there anything else you would like to say about the MMI or other aspects of the selection process?
